# Supplementary material for: Convalescent COVID-19 patients are susceptible to endothelial dysfunction due to persistent immune activation
Source: eLife. 2021 Mar 23;10:e64909. doi: 10.7554/eLife.64909 (PMC7987341; doi:10.7554/eLife.64909)
Supplement: Supplementary file 1. [file elife-64909-supp1.docx]

## Supplementary File 1

Title: Statistical adjustment for age

Description: Analysis of plasma cytokine levels (a) and correlative studies of plasma cytokines and number of CECs (b) after adjustment for age factor.

|  | **β (95% CI)** | ***p* value** |
| --- | --- | --- |
| Age | 12.33 (3.83-20.83) | **0.006** |
| **Early acute phase** | | |
| BDNF | *−18.16 (−45.07-8.75) | 0.176 |
| PDGF-BB | *−30.98 (−133.80-71.80) | 0.539 |
| PIGF-1 | *18.56 (−664.20-701.30) | 0.956 |
| IL-1β | *−1.56 (−4.14-1.02) | 0.225 |
| **Early convalescent phase** | | |
| IL-1β | *2.39 (−0.92-5.71) | 0.150 |
| IL-17A | *5.11 (−6.39-16.61) | 0.370 |
| IL-2 | *32.92 (−1.88-67.72) | 0.062 |
| RANTES | *21.18 (0.46-41.90) | **0.045** |

Bold indicates statistical significance at alpha level 0.05. ^a^ Linear regression models with plasma cytokines were adjusted for age.

1. Partial correlation of plasma cytokines and number of CECs after controlling for age.

| **Correlated variables from Figure 3** | | **Partial correlation coefficient (*r*)** | ***p* value*** | **Test statistic** |
| --- | --- | --- | --- | --- |
| ***Convalescent COVID with CV risks*** | | | | |
| MIP-1α | CEC | 0.595 | **0.032** | 2.457 |
| IL-17A | SELP+ CEC | 0.484 | 0.094 | 1.836 |
| IL-8 | CX3CL1+ CEC | 0.614 | **0.026** | 2.580 |
| IL-18 | CX3CL1+ CEC | 0.591 | **0.033** | 2.432 |
| ***Convalescent COVID without CV risks*** | | | | |
| EGF | CEC | 0.660 | **0.020** | 2.778 |
| LIF | CEC | 0.800 | **0.002** | 4.210 |
| PDGF-BB | SELP+ CEC | 0.672 | **0.017** | 2.869 |
| PIGF-1 | CX3CL1+ CEC | 0.635 | **0.026** | 2.600 |
| IL-1RA | CEC | 0.546 | 0.066 | 2.062 |
| IP-10 | CEC | 0.706 | **0.010** | 3.152 |
| CXCL12 | CEC | 0.631 | **0.028** | 2.571 |
| RANTES | SELP+ CEC | 0.456 | 0.136 | 1.622 |
| IL-5 | CEC | 0.734 | **0.007** | 3.418 |
| IL-7 | SELP+ CEC | 0.753 | **0.005** | 3.623 |
| IL-18 | SELP+ CEC | 0.547 | 0.066 | 2.066 |
| IL-4 | CX3CL1+ CEC | 0.548 | 0.065 | 2.070 |

*Bold *p* values indicate statistical significance at alpha level 0.05 with non-parametric partial correlation coefficients calculated based on Spearman’s rank correlation. n= 14 for Convalescent COVID with CV risks and n= 13 for Convalescent COVID without CV risks.
